# Supplementary material for: Distinct representations of basic taste qualities in human gustatory cortex
Source: Nat Commun. 2019 Mar 5;10:1048. doi: 10.1038/s41467-019-08857-z (PMC6401093; doi:10.1038/s41467-019-08857-z)
Supplement: Supplementary file 1 — Supplementary Information [file 41467_2019_8857_MOESM1_ESM.pdf]

Supplementary Information

Distinct representations of basic taste qualities in human gustatory cortex

Junichi Chikazoe, Daniel H. Lee, Nikolaus Kriegeskorte, Adam K. Anderson

Number of figures: 4

Number of tables: 4

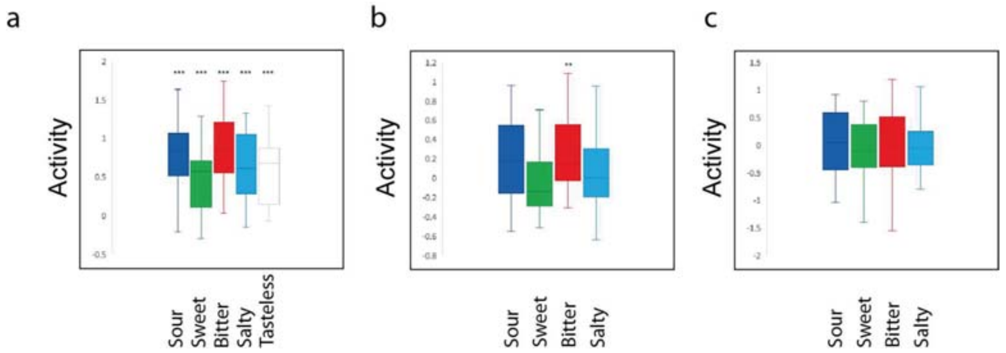

Supplementary Figure 1. Insula activity shows bitter sensitivity relative to tasteless without controlling for valence. a. Average voxel activity for each taste against baseline **b.** Average voxel activity for each taste against tasteless solution. **c.** Average voxel activity for each taste against tasteless solution with valence regressed out. Boxes represent the median and 25th/75th percentiles, and whiskers represent the minimum and maximum. ** $p < .01$, ***$p < .001$ uncorrected

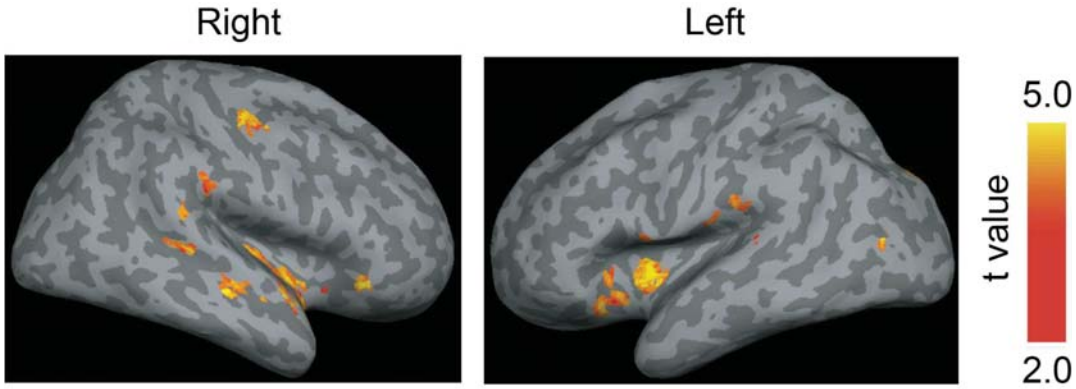

Supplementary Figure 2. Whole brain activation map for bitter vs. tasteless without controlling for valence. This map demonstrates the bitter-related insular activation reported by meta-analysis[15], suggesting sufficient strength of the gustatory signal to detect single tastes. Map thresholded at FDR < 5%.

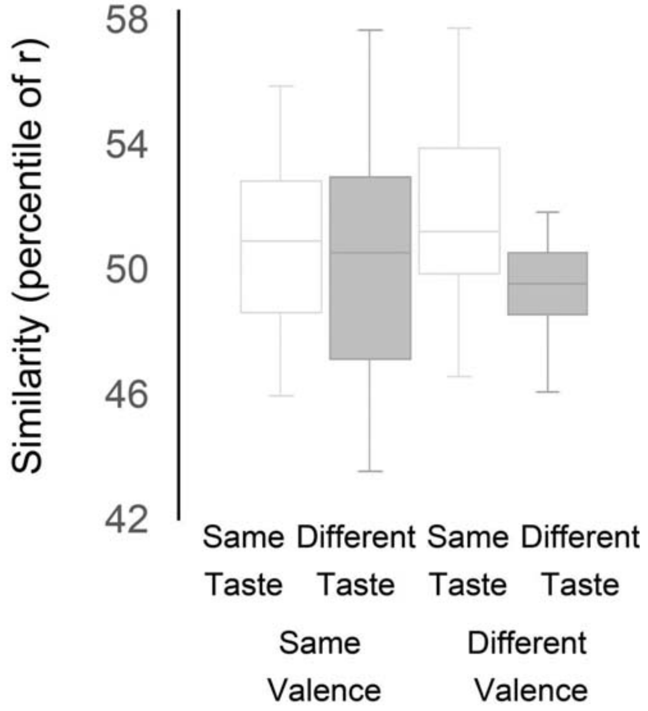

Supplementary Figure 3. Valence and taste type separability analysis without data dependency. To ensure no data dependency in the valence and taste type analysis, we conducted a separate repeated measures ANOVA on non-overlapping sets of trials for each of the 2 × 2 cells of taste type and valence (where the dependent variable was trial-by-trial similarity measured as correlations). This similarly resulted in a main effect of taste type ($F_{(1,19)} = 5.6$, $p = .028$), but no main effect of valence and no interaction, suggesting taste types are discriminable separate from their valence content. Boxes represent the median and 25th/75th percentiles, and whiskers represent the minimum and maximum.

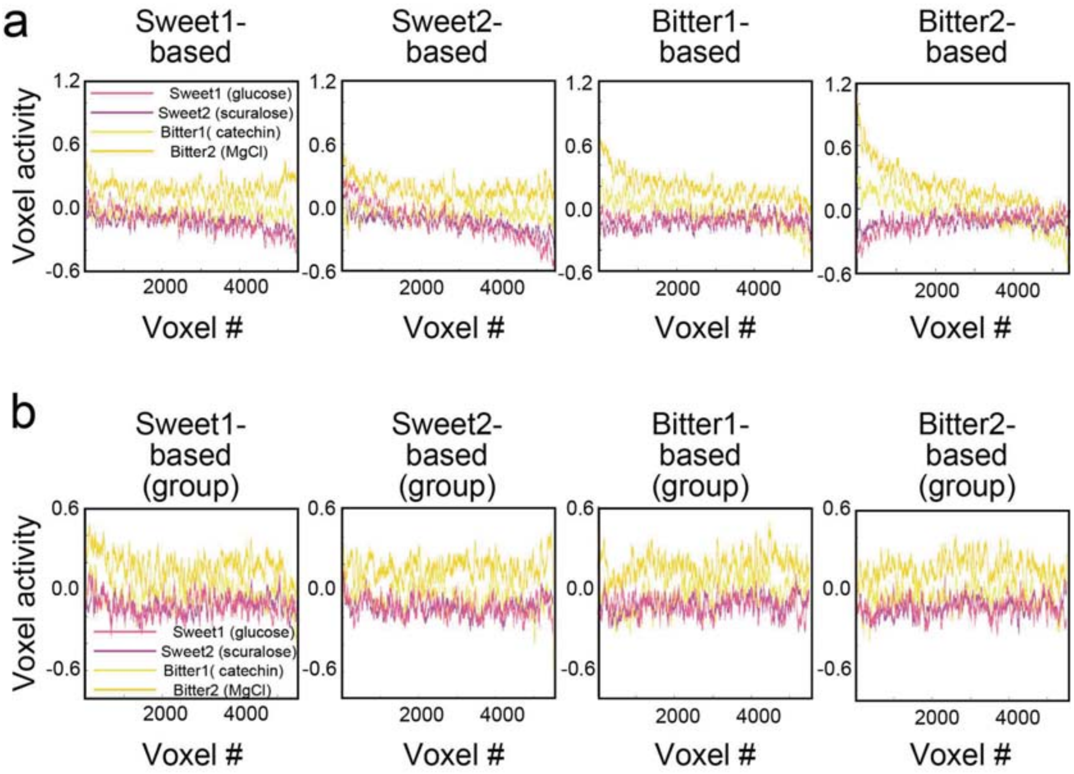

Supplementary Figure 4. Rank-ordered voxel sensitivity across taste stimuli under super-high field strength. a. Each participant's insular voxel activity to each taste stimulus in odd runs were rank-ordered to each taste in even runs based on sensitivity, then averaged across participants. The downward trend for matching taste types suggest voxel-specific taste tuning within subjects detected at higher resolution (also see Fig 4a). **b.** The same analyses as (a) but each participant's voxels activity to each taste stimulus in were aligned to each taste for all remaining participants based on sensitivity. The lack of downward trends suggest no voxel-specific taste tuning at the group level (also see

Fig 4c). Sweet 1: glucose, Sweet 2: sucralose, Bitter 1: catechin, and Bitter 2: magnesium chloride.

Supplementary Table 1. Valence variability within subject. The value in each cell indicates the proportion (%) of trials.

Taste type	Relative valence within subject										
	-5	-4	-3	-2	-1	0 (Median)	+1	+2	+3	+4	+5
Sour	0.3	0.5	2.8	5.1	17.9	46.7	14.1	5.6	5.3	0.8	1.0
Sweet	0.0	1.0	2.0	6.8	21.7	50.8	11.6	2.3	1.3	2.5	0.0
Bitter	0.0	2.0	1.3	6.3	18.9	49.2	12.1	7.8	1.5	0.0	0.8
Salty	0.0	0.0	1.3	6.6	20.5	43.7	15.2	7.1	3.0	2.3	0.5
Tasteless	0.3	0.5	1.8	7.3	17.2	55.8	13.1	3.5	0.5	0.0	0.0

Supplementary Table 2. Summary of univariate analysis for bitter vs. tasteless without valence regression.

Anatomic Region	Side	X	Y	Z	*t*-value
Insula	L	-38	4	-4	6.9
Medial superior frontal gyrus	L	-2	42	22	6.4
Insula	L	-32	6	2	6.2
Caudate	L	-6	6	2	6.0
Anterior cingulate gyrus	R	4	18	22	5.9
Insula/Superior temporal gyrus	R	40	4	-16	5.8
Insula/Superior temporal gyrus	R	38	-4	-10	5.7
Insula/Inferior frontal operculum	R	44	20	-6	5.7
Middle temporal gyrus	R	54	-26	-8	5.7
Supplementary motor area	L/R	0	6	64	5.4
Anterior cingulate gyrus	R	6	28	22	5.3
Anterior cingulate gyrus	L	-10	36	18	5.2
Rolandic operculum	L	-48	6	0	4.9
Anterior cingulate gyrus	L	-2	12	28	4.6
Middle cingulate gyrus	R	2	-34	52	4.2
Caudate	L	-14	-4	18	4.1
Middle cingulate gyrus	R	6	22	30	4.1
Thalamus	L	-4	-20	0	4.1
Thalamus	R	6	-20	4	4.0
Caudate	R	10	8	8	5.2
Caudate	R	6	2	0	4.6
Middle cingulate gyrus	R	8	-10	46	4.6
Middle occipital cortex	L	-50	-76	12	4.5
Supramarginal gyrus	R	64	-30	32	4.4

Supplementary Table 3. Summary of four-taste overlap revealed by multivoxel pattern analysis.

					t-value			
Anatomic Region	Side	X	Y	Z	Sour	Sweet	Bitter	Salty
Insula	L	-46	6	0	6.3	7.4	6.7	9.0
Insula	R	38	22	0	7.2	7.9	7.2	6.7
Precentral gyrus	R	50	-2	48	6.9	7.0	6.7	7.1
Heschl's gyrus	R	52	-8	4	8.6	7.2	5.2	6.8
Rolandic operculum/Frontal operculum	R	54	12	0	5.4	9.6	6.1	6.8
Precentral gyrus	R	56	-8	42	8.0	7.1	6.1	5.8
Vermis 8	R	2	-62	-34	5.9	7.3	7.1	6.5
Middle temporal gyrus	R	60	-4	-18	6.0	6.4	7.6	6.5
Rolandic operculum	R	50	-24	18	6.0	7.8	5.9	6.6
Supramarginal gyrus	R	60	-22	24	5.7	5.8	6.8	8.1
Lingual gyrus	R	2	-70	8	6.2	6.6	5.4	6.4

Supplementary Table 4. Proportion of combinations of same/different taste type with same/different valence trials.

		Different Taste Type	Same Taste Type
Full Dataset	Different Valence	74.2 ± 4.1 %	12.6 ± 2.5 %
	Same Valence	6.6 ± 4.1 %	6.7 ± 2.5 %
Independent Dataset	Different Valence	5.6 ±1.4 %	1.2 ±0.3 %
	Same Valence	0.8 ±0.4 %	0.8 ±0.3 %
