# Supplementary material for: Distinct representations of basic taste qualities in human gustatory cortex
Source: Nat Commun. 2019 Mar 5;10:1048. doi: 10.1038/s41467-019-08857-z (PMC6401093; doi:10.1038/s41467-019-08857-z)
Supplement: Supplementary file 3 — Reporting Summary [file 41467_2019_8857_MOESM3_ESM.pdf]

nature research

Corresponding author(s): Junichi Chikazoe, Adam K. Anderson

Reporting Summary

Nature Research wishes to improve the reproducibility of the work that we publish. This form provides structure for consistency and transparency in reporting. For further information on Nature Research policies, see Authors & Referees and the Editorial Policy Checklist.

Statistical parameters

When statistical analyses are reported, confirm that the following items are present in the relevant location (e.g. figure legend, table legend, main text, or Methods section).

n/a	Confirmed	
☐	☒	The exact sample size (n) for each experimental group/condition, given as a discrete number and unit of measurement
☐	☒	An indication of whether measurements were taken from distinct samples or whether the same sample was measured repeatedly
☐	☒	The statistical test(s) used AND whether they are one- or two-sided<br>*Only common tests should be described solely by name; describe more complex techniques in the Methods section.*
☐	☒	A description of all covariates tested
☐	☒	A description of any assumptions or corrections, such as tests of normality and adjustment for multiple comparisons
☐	☒	A full description of the statistics including central tendency (e.g. means) or other basic estimates (e.g. regression coefficient) AND variation (e.g. standard deviation) or associated estimates of uncertainty (e.g. confidence intervals)
☐	☒	For null hypothesis testing, the test statistic (e.g. F, t, r) with confidence intervals, effect sizes, degrees of freedom and P value noted<br>*Give P values as exact values whenever suitable.*
☒	☐	For Bayesian analysis, information on the choice of priors and Markov chain Monte Carlo settings
☒	☐	For hierarchical and complex designs, identification of the appropriate level for tests and full reporting of outcomes
☒	☐	Estimates of effect sizes (e.g. Cohen's d, Pearson's r), indicating how they were calculated
☐	☒	Clearly defined error bars<br>*State explicitly what error bars represent (e.g. SD, SE, CI)*

Our web collection on statistics for biologists may be useful.

Software and code

Policy information about availability of computer code

Data collection	N/A
Data analysis	Data were analyzed using SPM8 software (http://www.fil.ion.ucl.ac.uk/spm/). To visualize the imaging results, freesurfer software (http://surfer.nmr.mgh.harvard.edu/) and SPM surfrend toolbox (written by I. Kahn; http://spmsurfrend.sourceforge.net) were used after modification.

For manuscripts utilizing custom algorithms or software that are central to the research but not yet described in published literature, software must be made available to editors/reviewers upon request. We strongly encourage code deposition in a community repository (e.g. GitHub). See the Nature Research guidelines for submitting code & software for further information.

Data

Policy information about availability of data

All manuscripts must include a data availability statement. This statement should provide the following information, where applicable:
- Accession codes, unique identifiers, or web links for publicly available datasets
- A list of figures that have associated raw data
- A description of any restrictions on data availability

All relevant data and code used to generate results are available from the authors on request.

April 2018

Field-specific reporting

Please select the best fit for your research. If you are not sure, read the appropriate sections before making your selection.

☒ Life sciences ☐ Behavioural & social sciences ☐ Ecological, evolutionary & environmental sciences

For a reference copy of the document with all sections, see nature.com/authors/policies/ReportingSummary-flat.pdf

Life sciences study design

All studies must disclose on these points even when the disclosure is negative.

Sample size	No statistical test was run to determine sample size a priori. The sample sizes we chose are similar to those used in previous publications.
Data exclusions	N/A
Replication	Using 3T and 7T MRI scanners, we relplicated multi-taste reprsentations in the insula, which were measured in terms of classification performance for taste type.
Randomization	N/A
Blinding	N/A

Reporting for specific materials, systems and methods

Materials & experimental systems

n/a	Involved in the study
☒	☐ Unique biological materials
☒	☐ Antibodies
☒	☐ Eukaryotic cell lines
☒	☐ Palaeontology
☒	☐ Animals and other organisms
☐	☒ Human research participants

Methods

n/a	Involved in the study
☒	☐ ChIP-seq
☒	☐ Flow cytometry
☐	☒ MRI-based neuroimaging

Human research participants

Policy information about studies involving human research participants

Population characteristics	Experiment 1:<br>Twenty healthy adults (11 male, ages 26.2 ± 3.1, Canadian, no history of psychiatric disorder)<br><br>Experiment 2:<br>Eleven healthy adults (6 male, ages 22.2 ± 2.2, Japanese, no history of psychiatric disorder)
Recruitment	Participants were recruited through printed and electronic advertisements. No biases were expected.

Magnetic resonance imaging

Experimental design

Design type	task; event-related
Design specifications	Experiment 1:<br>100 taste solution trials were randomized and balanced across five runs. In each trial, 0.5 mL of taste solution was delivered over 1244 ms. When liquid delivery ended, a screen instructed participants to swallow the liquid (1 s). After 7756 ms, scaling bars appeared to rate positivity (3 s) then negativity (3 s) of the liquid. This was followed by 0.5 mL of the tasteless liquid delivery during 1244 ms for rinsing, followed by the 1 s swallow instruction. After a 7756 ms inter-trial-interval, the next trial began.<br><br>Experiment 2:

100 taste solution trials were randomized and balanced across five runs. In each trial, 0.88 mL of taste solution was delivered over 2 s. When liquid delivery ended, a screen instructed participants to swallow the liquid (2 s). After 4000 ms, scaling bars appeared to rate positivity (3 s) then negativity (3 s) of the liquid. This was followed by 0.88 mL of the tasteless liquid delivery during 2 s for rinsing, followed by the 2 s swallow instruction. After a 7 s inter-trial-interval, the next trial began.

Behavioral performance measures

After each taste trial, participants rated the experience along two independent sliding scales for positive (pleasant) and negative (unpleasant) hedonic valence.

Acquisition

Imaging type(s)

functional

Field strength

3T for Experiment 1, 7T for Experiment 2

Sequence & imaging parameters

Experiment 1 (3T):
Localizer images were first collected to align the field of view centered on each participant's brain. T1-weighted anatomical images were obtained (1 mm3, 256 × 256 FOV; MPRAGE sequence) before the experimental EPI runs. For functional imaging, a gradient echo-planar sequence was used (TR = 2000 ms; TE = 27 ms; flip angle = 70 degrees). Each functional run consisted of 263 whole brain acquisitions (40 × 3.5 mm slices; interleaved acquisition; field of view = 192 mm; matrix size = 64 × 64; in-plane resolution of 3 mm).

Experiment 2 (7T):
Localizer images were first collected to align the field of view centered on each participant's brain. T1-weighted anatomical images were obtained (0.75mm isometric, 224 × 300 FOV; MPRAGE sequence). For functional imaging, a gradient echo-planar sequence was used (TR = 500 ms; TE = 25 ms; flip angle = 35 degrees; multiband factor = 4). Each functional run consisted of 1010 whole brain acquisitions (32 × 2.0 mm slices; interleaved acquisition; field of view = 208 mm; matrix size = 104 × 104; in-plane resolution of 2 mm).

Area of acquisition

A whole brain in Experiment 1, planes covering the insula in Experiment 2

Diffusion MRI ☐ Used ☒ Not used

Preprocessing

Preprocessing software

Data were analyzed using SPM8 software (http://www.fil.ion.ucl.ac.uk/spm/). Functional images were realigned, slice timing corrected, and normalized to the MNI template (ICBM 152) with interpolation to a 2 × 2 × 2 mm space. Data was spatially smoothed (full width, half maximum = 6mm) for univariate parametric modulation analysis but not for MVPA since it may impair MVPA performance.

Normalization

Linear (12-parameter affine transformation) and nonlinear (warping) registration were used. A structural image was used for normalization.

Normalization template

ICBM152

Noise and artifact removal

Motion artifact was removed by adding motion regressors to the design matrix at the 1st level analysis.

Volume censoring

Volume censoring was not applied.

Statistical modeling & inference

Model type and settings

Taste type effects were analyzed by univariate and multivariate analysis. For univariate analysis, regressors coding each tastant were time-locked to stimulus presentation. For multivariate analysis, each stimulus presentation was modeled as a separate event, using canonical function.

Effect(s) tested

Effect of taste stimuli (sour; salty; bitter; sweet) was tested. To dissociate effect of taste type from valence, an ANOVA was used.

Specify type of analysis: ☐ Whole brain ☐ ROI-based ☒ Both

Anatomical location(s)

The insula is known as the putative gustatory cortex. We defined the insula, based on anatomical information (AAL: Automated Anatomical Labeling).

Statistic type for inference
(See Eklund et al. 2016)

Voxel-wise (small volume correction/ whole brain)

Correction

FWE, FDR, permutaion

Models & analysis

n/a	Involved in the study
☒	☐ Functional and/or effective connectivity
☒	☐ Graph analysis
☐	☒ Multivariate modeling or predictive analysis

Multivariate modeling and predictive analysis

We analyzed fMRI data using searchlight (radius of 4 mm, including 33 voxels) analysis. Within a given sphere for each participant, a vector was created containing the spatial pattern of BOLD-MRI signal time-locked to stimulus presentation (normalized t-values per voxel). To evaluate whether the activity patterns in the searchlight spheres are capable of discriminating taste types, we employed a leave-one-stimulus-pair-out cross-validation. In this procedure, the linear discriminant analysis (LDA) classifier was trained on 38 trials which included the tested taste type and another taste type (19 trials for each) and then tested on the left-out stimulus pair.
